# The Hierarchical Organization of the Default, Dorsal Attention and Salience Networks in Adolescents and Young Adults

**DOI:** 10.1093/cercor/bhx307

**Published:** 2017-11-17

**Authors:** Yuan Zhou, Karl J Friston, Peter Zeidman, Jie Chen, Shu Li, Adeel Razi

**Affiliations:** 1 CAS Key Laboratory of Behavioral Science, Institute of Psychology, Beijing 100101, China; 2 Magnetic Resonance Imaging Research Center, Institute of Psychology, Chinese Academy of Sciences, Beijing 100101, China; 3 Department of Psychology, University of Chinese Academy of Sciences, Beijing 100049, China; 4 The Wellcome Trust Centre for Neuroimaging, University College London, Queen Square, London WC1N 3BG, UK; 5 CAS Key Laboratory of Mental Health, Institute of Psychology, Beijing 100101, China; 6 Monash Biomedical Imaging and Monash Institute of Cognitive & Clinical Neurosciences, Monash University, Clayton 3800, Australia; 7 Department of Electronic Engineering, NED University of Engineering and Technology, Karachi 75270, Pakistan

**Keywords:** default network, dorsal attention network, dynamic causal modeling, resting-state fMRI, salience network

## Abstract

An important characteristic of spontaneous brain activity is the anticorrelation between the core default network (cDN) and the dorsal attention network (DAN) and the salience network (SN). This anticorrelation may constitute a key aspect of functional anatomy and is implicated in several brain disorders. We used dynamic causal modeling to assess the hypothesis that a causal hierarchy underlies this anticorrelation structure, using resting-state fMRI of healthy adolescent and young adults (*N* = 404). Our analysis revealed an asymmetric effective connectivity, such that the regions in the SN and DAN exerted an inhibitory influence on the cDN regions; whereas the cDN exerted an excitatory influence on the SN and DAN regions. The relative strength of efferent versus afferent connections places the SN at the apex of the hierarchy, suggesting that the SN modulates anticorrelated networks with descending hierarchical connections. In short, this study of directed neuronal coupling reveals a causal hierarchical architecture that generates or orchestrates anticorrelation of brain activity. These new findings shed light on functional integration of intrinsic brain networks at rest and speak to future dynamic causal modeling studies of large-scale networks.

## Introduction

The brain is organized into multiple distributed (large-scale) systems. An important aspect of endogenous or spontaneous activity is that a default network (DN), engaged during rest and internally directed tasks, exhibits anticorrelation with networks engaged during externally directed tasks, such as the dorsal attention network (DAN) and the salience network (SN) (Fox et al. 2005; Fransson 2005; Smith et al. 2009; Andrews-Hanna et al. 2014; Raichle 2015). The DN comprises multiple interacting subsystems (Andrews-Hanna et al. 2010b). Among of them, the core subsystem—comprising the posterior cingulate cortex (PCC) and medial prefrontal cortex (MPFC)—is implicated in self-referential mental activities (Buckner et al. 2008; Andrews-Hanna et al. 2010a; Raichle 2015). The DAN, which comprises the frontal eye field (FEF) and inferior parietal sulcus (IPS), is implicated in directed attention and working memory (Corbetta and Shulman 2002; Fox et al. 2006). The SN, which comprises the anterior insula (AI) and dorsal anterior cingulate cortex (dACC), is important for detection and mapping of external salient inputs and task control (Dosenbach et al. 2007; Seeley et al. 2007; Menon and Uddin2010; Uddin 2015). Although data preprocessing might introduce spurious anticorrelation (Murphy et al. 2009; Murphy and Fox 2016), the core subsystem of DN (cDN) shows reproducible negative correlations with the dorsal attention and SNs (Chen et al. 2017; Dixon et al. 2017). Furthermore, mounting evidence from human and animal studies support a biological basis for this anticorrelation. First, it has been shown that anticorrelation remains after global signal regression (Fox et al. 2009; Carbonell et al. 2014). Second, magnetoencephalography recordings of spontaneous activity have successfully characterized the anticorrelation at higher temporal resolution (Baker et al. 2014). Third, using detailed computer simulations of mammalian cerebral cortex, slow anticorrelated fluctuations of functional modes have been shown to emerge (Honey et al. 2007; Izhikevich and Edelman 2008; Deco et al. 2009). Finally, electrophysiological evidence in cats and human suggest a neuronal origin for anticorrelated fluctuations in the blood-oxygen level dependent (BOLD) signals (He et al. 2008; Popa et al. 2009). Therefore, the anticorrelation between the cDN and the dorsal attention and SNs may reflect a key aspect of functional integration of the brain. The communication and coordination between these intrinsic, anticorrelated networks are considered crucial for information integration and cognitive functioning (Williamson 2007; Jiang 2013).

However, a physiological understanding of how anticorrelated networks arise is still lacking. In theory, there are at least 2 possibilities for the interaction: one is that the dorsal attention and SNs negatively regulate the cDN; another one is that the cDN negatively regulates the dorsal attention and SNs. These possibilities cannot be tested using (symmetric) functional connectivity methods, because correlations do not reveal the directed causal influence of one neural system on another (Friston 2011). Effective connectivity methods, which explicitly test for directed causal influences between neural systems (Friston 1994, 2011), are well placed to reveal how anticorrelated networks are hierarchically organized in terms of feedforward (usually excitatory) and feedback (usually inhibitory) connections. In the existing literature, there are few attempts that assess interactions between the anticorrelated networks using directed measures of functional connectivity based on temporal precedence, for example, Granger causality. Using this method, Sridharan et al. (2008) found a dominant outflow from the right AI, a key region in the SN, to the PCC, the core DN region during rest. Meanwhile, other studies found that the regions in the DN may causally influence activity in anticorrelated networks (Uddin et al. 2009a) or provide evidence for bidirectional information flow between one of the key region in the DN (i.e., PCC) and the DAN (Deshpande et al. 2011). However, the interpretability of Granger causality analysis is questionable when applied to fMRI data because “lag-based” causality may be compromised by differences in hemodynamic lags between regions, in addition to the poor temporal resolution and measurement noise intrinsic to fMRI (Smith et al. 2011; Webb et al. 2013). Given the ambiguous nature of the current literature, we sought to understand the nature of these interactions using dynamic causal modeling (DCM) (Friston et al. 2003), which is better suited to disclose the causal and directed nature of coupling between intrinsic modes of brain activity.

Crucially, because DCM is based upon neuronal dynamics that are described with differential equations (a neuronal state space model), global fluctuations in brain activity cannot influence the assessment of effective connectivity. This is because the influence of one region over another is modeled in terms of rates of change of activity. In other words, globally coherent fluctuations (i.e., confounds) cannot be explained in terms of effective connectivity (because the responses elicited in a target are uncorrelated with the activity of a source region). DCM is therefore in a position to resolve debates about the confounding effects of globally coherent signals (or their removal) on measurements of functional connectivity and the mediation of anticorrelated activity. In this study, we use spectral DCM (Friston et al. 2014) and its recent extension to whole brain networks (Razi et al. 2017) to test the hypothesis that a hierarchy of directed connections can explain anticorrelation between intrinsic brain networks (Friston et al. 2003; Friston 2008).

Previously, DCM has been largely used to identify network structure based on fMRI time series data (Friston et al. 2011; Seghier and Friston 2013; Di and Biswal 2014). The recent development of spectral DCM, which operates in the frequency domain rather than the time domain, provides estimates of effective connectivity that underlie intrinsic functional connectivity during rest (Razi and Friston 2016). We applied spectral DCM in conjunction with a newly developed framework for group studies that uses parametric empirical Bayes (PEB) (Friston et al. 2016). We estimated the predominant causal connections between regions in 2 anticorrelated networks in the hope of understanding of how these anticorrelated networks interact with—or contextualize—each other. Finally, we provide an empirical illustration of the relationship between effective connectivity and functional connectivity—a relationship that is vital for understanding the functional interaction of large-scale brain networks.

## Methods

### Participants

Our sample was based on 420 adolescents and young adults from the Beijing Twin Study at the Institute of Psychology, Chinese Academy of Sciences (Chen et al. 2013b). Because our objective in this study was to characterize commonalities across subjects, the use of twin-pairs was incidental to our hypotheses. However, this dataset provided an exceptionally large sample size, with the added advantage of reducing interindividual variability due to genetic and common environmental factors. After excluding 8 twins pairs who were outliers in terms of unusually large head motion (for details, please see Preprocessing), 202 same-sex twin pairs (111 monozygotic and 91 dizygotic twin pairs; mean age: 17.4 ± 2.1 years, age range: 14–23 years; 48.5% females) were included in the final analyses. Written informed consent was obtained from all participants or their guardians. This study was approved by the Institutional Review Board of the Institute of Psychology of the Chinese Academy of Sciences and the Institutional Review Board of Beijing MRI Center for Brain Research.

### Data Acquisition

The MRI data were acquired with a 3.0-T Siemens MRI scanner (MAGNETOM TRIO) in the Beijing MRI Center for Brain Research. Whole-brain rsfMRI scans were collected in 32 axial slices using an echo-planar imaging (EPI) sequence (repetition time [TR] = 2000 ms, echo time [TE] = 30 ms; flip angle [FA] = 90°, matrix = 64 × 64; field of view [FoV] = 220 × 220 mm^2^; slice thickness = 3 mm; slice gap = 1 mm). Each fMRI session lasted 6 min and thus contained 180 volumes. During the rsfMRI acquisition, the participants were explicitly instructed to lie supine, stay relaxed with their eyes closed, and move as little as possible. High-resolution T1-weighted images were acquired in a sagittal orientation using a magnetization-prepared rapid gradient-echo (MPRAGE) sequence (TR/TE = 2530/3.37 ms; FA = 7°; FoV = 240 mm^2^; 1-mm in-plane resolution; slice thickness = 1.33 mm, no gap; 144 slices).

### Preprocessing

Conventional functional imaging preprocessing was performed using SPM12 (revision 6750, www.fil.ion.ucl.ac.uk/spm) and Data Processing Assistant for Resting-State fMRI (DPARSF 4.1, http://www.restfmri.net), including the removal of the first 10 volumes, realignment, spatial normalization with 3-mm cubic voxels, a spatial smoothing of 6 mm FWHM and nuisance variable regression. The nuisance variables include 24 motion parameters (6 head motion parameters, 6 head motion parameters one time point before, and the 12 corresponding squared items), the signal averaged over the individual segmented CSF and white matter (WM) regions, linear and quadratic trends (Yan et al. 2013b). The rationale for including 24 motion parameters (hereafter “Friston-24”) as covariates is based on a comprehensive study, which assessed the impact of head micromovement on functional connectomics using several approaches from literature (Yan et al. 2013a). This study found that all the approaches considered demonstrated suppressed motion–BOLD relationships; however, the Friston-24 covariates showed the greatest reductions in both positive and negative motion–BOLD relationships. In addition, the Friston-24 approach produced the least motion-related spikes, when examining the BOLD signal after head motion correction (Yan et al. 2013a). This set of nuisance variables incidentally removes low-frequency fluctuations normally associated with global confounds.

The volume-based frame-wise displacement (FD) was used to quantify head motion (Power et al. 2012; Satterthwaite et al. 2012; Van Dijk et al. 2012). Outliers in head motion were identified with a mean FD larger than 3 interquartile ranges from the sample median.

### Selection and Extraction of Volumes of Interest

**Figure 1.**
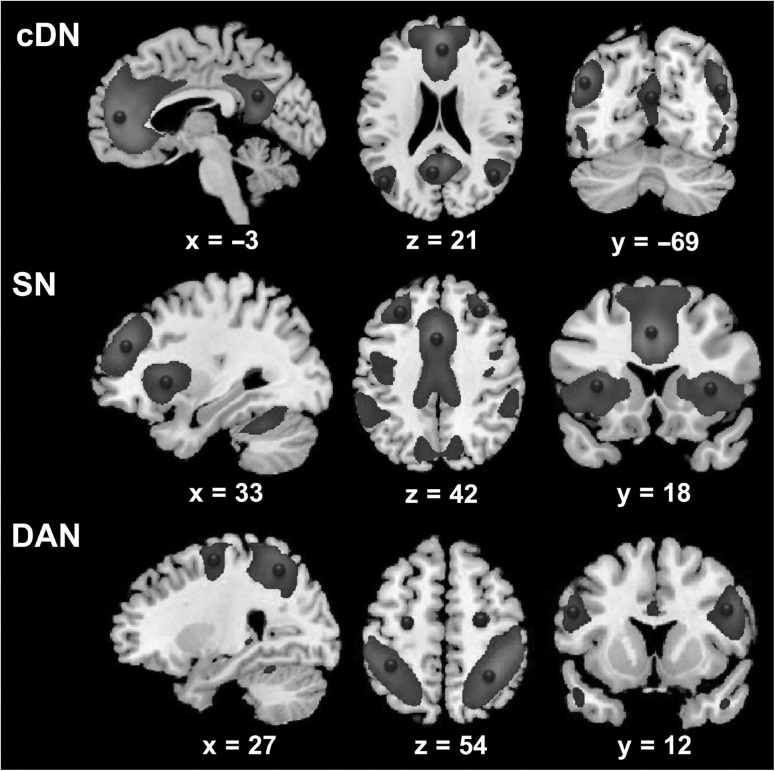
VOIs identified using spatial independent component analysis (ICA). The VOIs (circles) are overlaid on the spatial distribution maps derived from group ICA of 3 networks of interest, that is, the core default network (cDN), the salience network (SN), and the dorsal attention network (DAN).

The locations of the key cortical regions in each intrinsic network were identified with spatial ICA, as implemented in the Group ICA for fMRI Toolbox (GIFT, http://mialab.mrn.org/software/gift) (Calhoun et al. 2001). We extracted 20 components (Biswal et al. 2010; Shirer et al. 2012; Tsvetanov et al. 2016) from the preprocessed rsfMRI data. The 3 key intrinsic networks were identified by spatially matching with pre-existing templates (Shirer et al. 2012). The SN comprised five nodes: the dorsal cingulate cortex (dACC), the right and left AI (rAI/lAI), and the left and right anterior prefrontal cortex (aPFC). The DAN comprised 6 nodes: the right and left FEF (rFEF/lFEF), the left and right IFG and the right and left superior parietal lobes (rSPL/lSPL). For the core DN (cDN), we focused on 4 regions: the anterior MPFC (aMPFC), the PCC, the left and right angular gyrus (lAG and rAG). These regions were selected because they constitute a core part of DN (Andrews-Hanna et al. 2010b; Dixon et al. 2017) and most consistently showed anticorrelation with the dorsal attention and SNs (Fransson 2005; Fox et al. 2009; Uddin et al. 2009b; Chen et al. 2017; Dixon et al. 2017) The group-level regions and their peak coordinates are listed in Table 1 and are also shown in Figure 1.

**Table 1:** Locations of group-level volume of interest

Regions	MNI coordinates			Network
	*x*	*y*	*z*	
PCC	−3	−57	21	cDN
aMPFC	3	54	18	cDN
lAG	−48	−69	33	cDN
rAG	51	−63	27	cDN
dACC	−3	15	42	SN
lAI	−36	15	6	SN
rAI	33	18	6	SN
laPFC	−27	45	30	SN
raPFC	30	42	30	SN
lFEF	−24	−9	57	DAN
rFEF	27	−3	54	DAN
lIFG	−51	9	27	DAN
rIFG	54	12	30	DAN
lIPS	−42	−36	45	DAN
rIPS	39	−42	51	DAN

Abbreviation: l, left; r, right; PCC, posterior cingulate cortex; aMPFC, anterior medial prefrontal cortex; AG, angular gyrus; dACC, dorsal anterior cingulate cortex; AI, anterior insula; aPFC, anterior prefrontal cortex; FEF, frontal eye field; IFG, inferior frontal gyrus; IPS, inferior parietal sulcus; cDN, core default network; SN, salience network; DAN, dorsal attention network.

To identify subject-specific volumes of interest (VOI), we first obtained the group-level peak coordinates for each VOI—as outlined above—after correcting for the influence of interindividual differences by including age, gender, zygosity, and head motion as covariates in the GLM. Then, we obtained subject-specific, back-reconstructed, independent components spatial maps using the procedure implemented in the GIFT Toolbox (Calhoun et al. 2001). Subject-specific coordinates were identified as the peaks in subject-specific ICA maps within 8 mm of the group-level coordinates. Finally, we summarized regional time series with the principal eigenvariate of all (confound corrected) voxels within 8 mm of the subject-specific coordinates (and within the group mask). These time series were then used in subsequent DCM analysis. This specification of subject-specific VOI is summarized in the Supplementary Figure S1.

### Specification and Inversion of DCM at the First Level

The spectral DCM analyses were conducted using DCM12 implemented in the SPM12 (revision 6800, www.fil.ion.ucl.ac.uk/spm). For each participant, a fully connected model was created to compare all possible nested models of between- and within-network interactions (Friston et al. 2016). The DCM was estimated using spectral DCM, which fits the complex cross-spectral density using a power-law model of endogenous neuronal fluctuations (Friston et al. 2014; Razi et al. 2015). In order to avoid potential problems with overfitting in large-scale networks, which entail many free parameters—and to speed up the model inversion by suppressing conditional dependencies among parameters—we used functional connectivity to furnish priors on effective connectivity as described in (Seghier and Friston 2013; Razi et al. 2017). For more details, please see the text in the Supplementary Materials.

### Second Level Analysis Using Bayesian Model Reduction and Parametric Empirical Bayes

We used Parametric Empirical Bayes (PEB)—a between-subjects hierarchical or empirical Bayesian model over parameters—which models how individual (within-subject) connections relate to group or condition means. This hierarchical model treats intrinsic connectivity as a random (between-subjects) effect, which is modeled by adding a random Gaussian component to subject-specific parameters; that is, a general linear model of between subject effects generates the parameters of a within subject nonlinear (dynamic causal) model. This random effects modeling is important because, unlike a classical test (e.g., *t*-test), it uses the full posterior density over the parameters from each subject’s DCM—both the expected strength of each connection and the associated uncertainty (i.e., posterior covariance)—to inform the group-level result (i.e., group means).

To evaluate how regions in the anticorrelated networks interact, we used Bayesian model comparison to explore the space of possible hypotheses (or models), where each hypothesis assumed that a different combination of the connectivity parameters could characterize all the participants. Candidate models were obtained by removing one or more connections to produce nested or reduced forms of the full model. With 225 (15 regions times 15 regions) intrinsic connections (or parameters) of the fully connected model, there are a huge number of possible nested models in the model space. To address this we used Bayesian model reduction (BMR) that enables the evidence and parameters of nested models to be derived from a full model in a matter of milliseconds, enabling an efficient (greedy) search of the model space by scoring (based on the log model-evidence or free energy) each reduced model. For details, see Friston et al. (2016) and the Supplementary Materials. The search algorithm used BMR to prune connection parameters from the full model, until there was no further improvement in model-evidence. The parameters of the best 256 models from this search procedure were then averaged, weighted by their model evidence (Bayesian Model Averaging). This Bayesian model average is reported in the results and figures except where otherwise specified.

### Hierarchical Clustering Analysis

In order to characterize the network components from the group-level DCM, a hierarchical clustering analysis was performed on the effective connectivity. A similar analysis was also applied to the functional connectivity. In the context of spectral DCM, functional connectivity constitutes the data features of interest. To summarize functional connectivity, we used the correlation; namely, the normalized cross covariance function at zero lag (which is equivalent to the normalized cross spectral density, over all frequencies).

### Hierarchical Organization of the Resting State Networks

To examine the hierarchical strength of each network, we first computed the mean between-network connection strength taking into account uncertainty in these estimates. To do this we computed Bayesian contrasts of the connections as follows. Given contrast vector c with one element for each of P parameters, as well as the posterior density, N(Μ,Σ); where, N is the multivariate normal distribution, $M_{P\times 1}$ are the expected values of the parameters and $\Sigma_{P\times P}$ is their covariance matrix, the expected value of the contrast is as follows:

$$\mu =c^{T}M.$$

And the variance (or uncertainty) of the contrast is as follows:

$$\sigma^{2}=c^{T}\Sigma c.$$

This works in exactly the same way as computing contrasts in classical statistics, except the Bayesian posterior is used instead of maximum likelihood estimates. For example, to find the mean of the first 4 parameters, where P=6, $c=[0.250.25\;0.25\;0.25\;0\;0{]}^{\text{T}}$. To compare the mean of the first 2 parameters against the second 2 parameters, the contrast would be $c=[0.5\;0.5-\;0.5-\;0.5\;0\;0{]}^{\text{T}}$. We used contrasts to compute the hierarchical strength of each network by computing the difference between its averaged efferent and afferent connections. This approach is similar to that used for analyzing hierarchical projections in the monkey brain (Goulas et al. 2014) and the hierarchical organization of the prefrontal cortex in humans (Nee and D’Esposito 2016).

## Results

### Effective Connectivity

**Figure 2.**
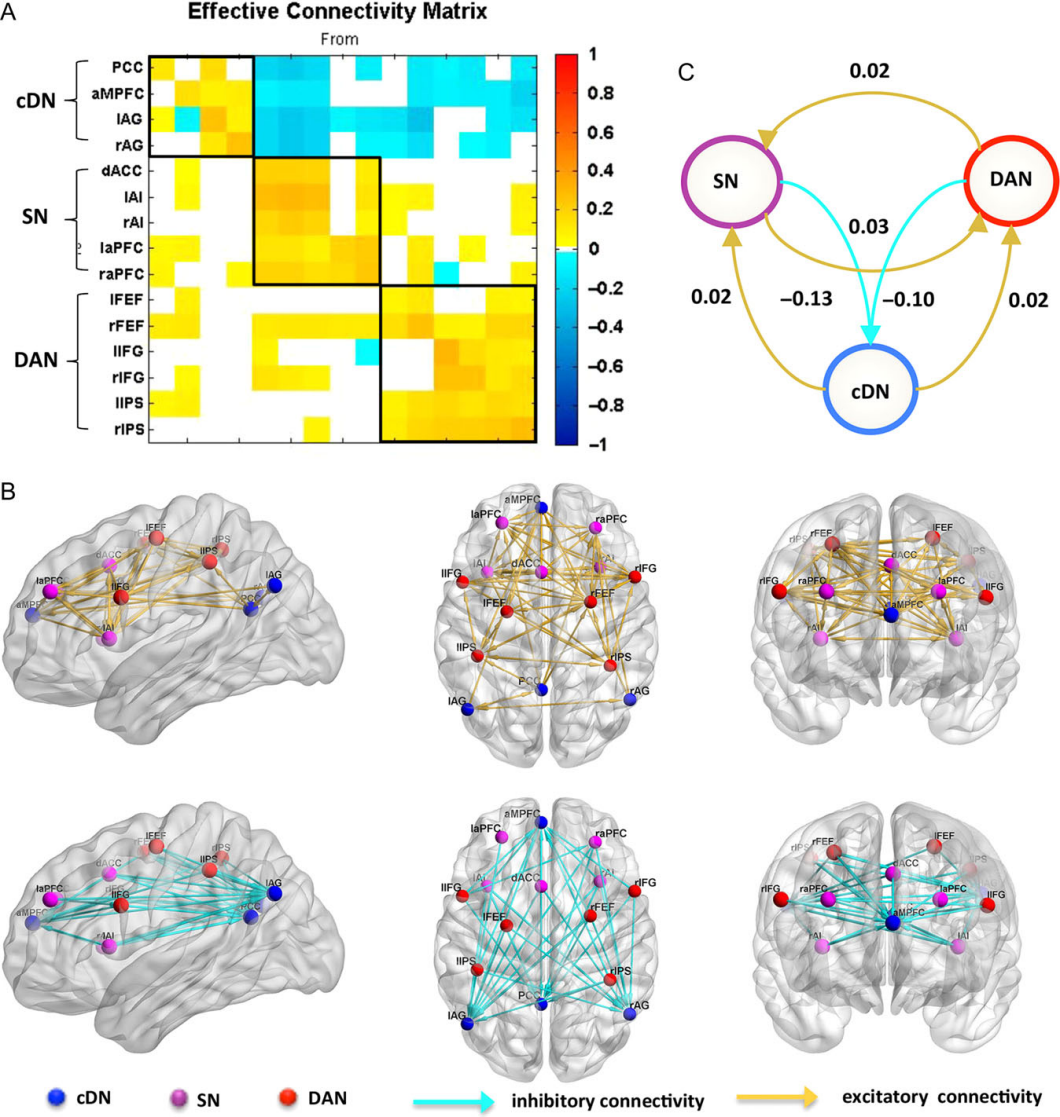
Effective connectivity within and between each network. (*A*) Effective connectivity matrix of the 15 brain regions after Bayesian Model Reduction (without any covariates). Connections were retained after pruning any parameters that did not contribute to the free energy (i.e., posterior probabilities with versus without parameter are larger than 95%). The color presents the connection parameters (in Hz) obtained by Bayesian Model Averaging (BMA). The 3 networks are highlighted using black lines. Note also the asymmetric, directional and sparse nature of the connectivity matrix. (*B*) The nodes and effective connections within and between each network have been mapped onto cortical surfaces using BrainNet Viewer software (http://www.nitrc.org/projects/bnv/). The effective connectivity reported here is the same as in (*A*). For visualization, we separated the inhibitory connectivity (cyan) from the excitatory (yellow). (*C*) A schematic summarizing effective connectivity between each network. This between network effective connectivity was calculated using Bayesian procedures (please see main text for details), which not only consider the connection strengths but also the conditional uncertainties (i.e., the covariance matrix). For visualization, we have separated the negative connections (cyan) from the positive (yellow). Abbreviations: please see Table 1.

The effective connectivity matrix obtained after group level analysis, using PEB estimation and BMR, is shown in Figure 2*A*. The effective connectivity matrix revealed the following main results: (1) regions belonging to the same network grouped together; (2) The connections originating from regions that belong to the salience and the DANs—and terminating in the cDN—were all negative, suggesting that these 2 networks inhibit activity in the cDN: in other words, the activity in the salience and DANs decreased the rate of change of activity in the DN; (3) most of the connections originating from the cDN regions and terminating in the salience and DANs were positive, suggesting the DN excites the activity in the 2 networks; and (4) there were bidirectional excitatory connections between the salience and DANs. The effective connectivity pattern is a mirror of the functional connectivity, in which negative connections between the cDN and the salience and DANs and positive connections within each network were found (compare Fig. 2*B* and Supplementary Fig. S2).

We repeated the above analysis with age, gender, zygosity and head motion as covariates in the (second level of the) PEB model to exclude the influence of interindividual differences. Pleasingly, we obtained a very similar effective connectivity pattern (Supplementary Fig. S3); establishing that the network architecture identify above was not explained by confounding between-subject effects. Although examining genetic factors was not the objective of this study, the fact that modeling whether subjects were zygotic twins or not suggests that this genetic factor did not have a large effect on the results. Similarly, age, sex and head motion have negligible effects on connectivity estimates (Supplementary Fig. S3).

We further validated the grouping of regions by networks with a hierarchical clustering analysis (Supplementary Fig. S4, left panel). The clustering analysis clearly identified the 2 modes, in which the cDN regions belong to one mode and the DAN and SN regions belong to another. Interestingly, the clustering pattern observed in effective connectivity is almost the same as that in functional connectivity (Supplementary Fig. S4, right panel).

Figure 2*C* shows the average effective connectivity between networks, illustrating a hierarchical structure among these 3 networks. For each network, the posterior probability that the averaged within-network connection differs from zero is (nearly) 100%. And the posterior probability that the averaged strength of connections from the cDN to the SN or DAN is different from the averaged strength of connections from the SN or DAN to the cDN is also (nearly) 100%. In addition, weak evidence for greater strength of connection from the SN to the DAN than that of reciprocal connections was found (posterior probability = 91%). These findings suggest a clear hierarchical structure among these 3 networks. That is, the SN and the DAN exerts an inhibitory influence on the activity of the cDN and the cDN exerts an excitatory influence on both of the SN and the DAN. We also computed the hierarchy strength, which is the difference between averaged unsigned efferent and afferent connection parameters between networks. The total hierarchy strength also validated the hierarchical structure among these 3 networks: the hierarchical strength of the SN (0.03 + |−0.13|−0.02 − 0.02 = 0.12) and the DAN (0.02 + | − 0.1| − 0.03 – 0.02 = 0.07) are greater than that of the cDN (0.02 + 0.02 − | − 0.13| − |−0.1| = −0.19).

### Mapping From Causes to Effects

**Figure 3.**
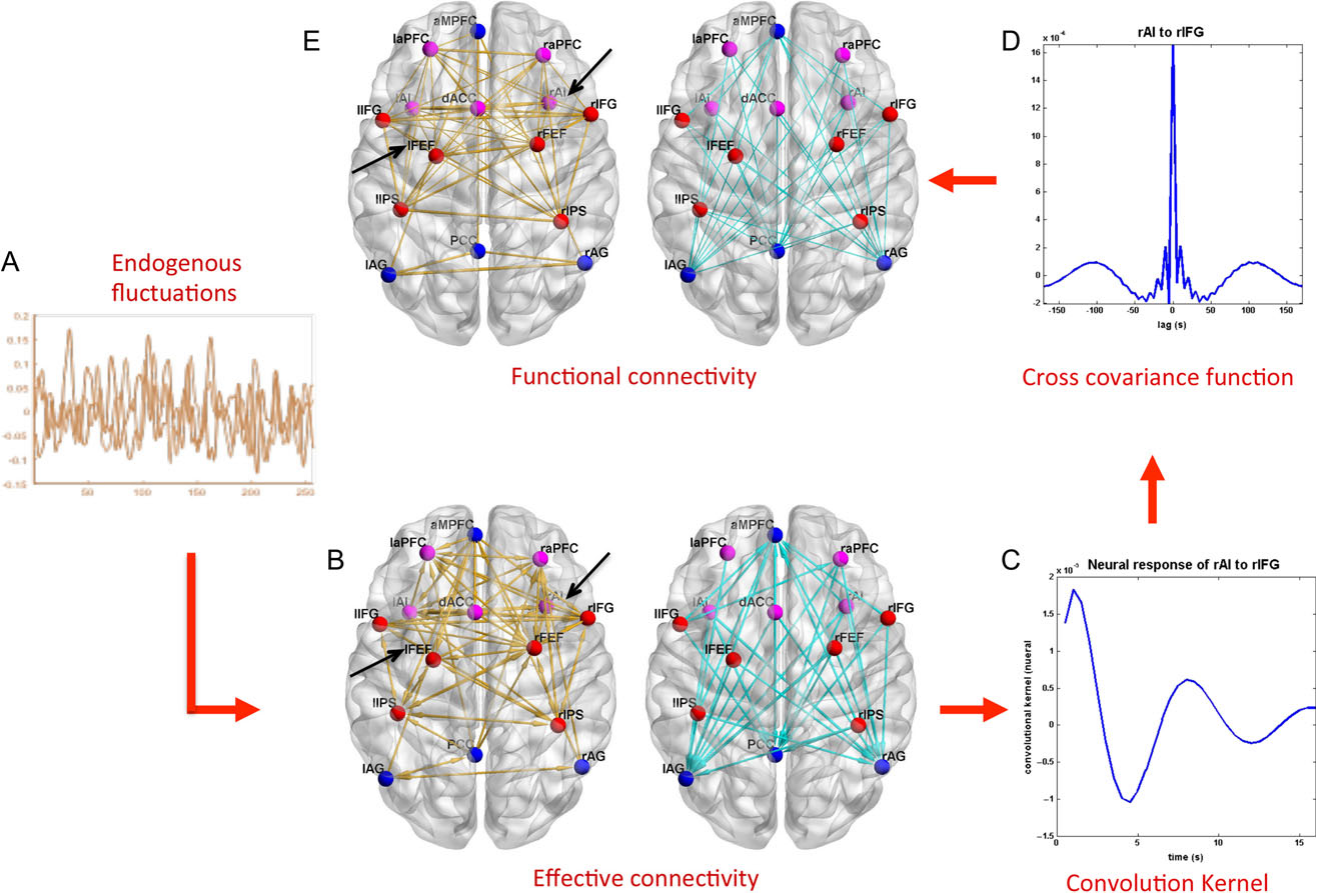
This schematic illustrates the forward (dynamic causal) model for modeling intrinsic or endogenous fluctuations. (*A*) Endogenous fluctuations in neural activity. (*B*) Effective connectivity after Bayesian Model Reduction (without covariates same as from previous figure). For visualization, we separated the inhibitory connectivity (cyan) from the excitatory connectivity (yellow). (*C*) An exemplar convolution kernel for one of the connections (averaged over participants). (*D*) Cross covariance function for the same connection as in (*C*). (*E*) Functional connectivity matrix computed from the cross-covariance function as Pearson correlations. Only significant connections were shown (FDR, *P* < 0.05). For visualization, we have separated the negative functional connectivity (cyan) from the positive (yellow). The connections indicated by black arrows highlight several interesting examples to show the differences between effective and functional connectivity (see Results and Discussion for details).

Figure 3 clarifies how the observations (functional connectivity) are caused by the underlying effective connectivity. The endogenous fluctuations (neural state noise, Fig. 3*A*) are the driving input that induces neural activity that in turn causes changes in the BOLD response, which together with observation noise forms the fMRI time series. This chain that links causes to effects is defined by a forward or generative model. Conversely, the discovery of causes from effects is an ill-posed problem—in the sense that there can be several patterns of effective connectivity that may cause the same observations (i.e., functional connectivity). The solution to this ill-posed problem requires model inversion that optimizes an objective function such as log-model evidence (or its proxy free energy) to finesse the ill-posed nature of this degenerate mapping (Friston et al. 2014; Razi et al. 2015).

To provide an empirical illustration of this mapping from causes (effective connectivity, Fig. 3*B*) to effects (functional connectivity, Fig. 3*E*), we show the intermediate steps that underlie the computation of a convolution kernel (averaged over all participants) in one representative connection (Fig. 3*C*) and in turn the cross-covariance as a function of time lags (Fig. 3*D*). The most common measures of functional connectivity—based on (Pearson) correlations—can easily be computed as zero-lag normalized cross co-variance (Fig. 3*E*); see Razi and Friston (2016) for more details.

The key insight here is that the effective and functional connectivity have a very different form. An important difference between effective and functional connectivity is that effective connectivity is generally much sparser. We highlight this by an example: the connection from the right IFG to the right AI is absent but the reciprocal connection (that is connection from the right AI to the right IFG) exists (indicated by an black arrow in Fig. 3*B*). However, when we look at the functional connectivity (calculated from the sampled time series) of right IFG with right AI, we see a positive correlation between these regions (indicated by a black arrow in Fig. 3*E*). Another example is in the connection with significant functional connectivity (e.g., left FEF to left IFG), where effective connectivity is absent (indicated by an arrow in Fig. 3*B*, *E*).

## Discussion

By capitalizing on recent developments in empirical Bayes (Friston et al. 2016) and spectral DCM, we characterized the effective connectivity between and within large-scale resting state networks that exhibit well-known anticorrelation between the core subsystem of DN and the salience and DANs. The 3 main findings of this work are: (1) using effective connectivity computed by spectral DCM, we could identify the brain networks that produce a canonical patterns of functional connectivity, (2) the between-network effective connectivity had an asymmetric hierarchy, such that the regions in the salience and the DAN showed inhibitory influence on the cDN; whereas the cDN exerted excitatory influence on the salience and the DANs, and (3) based on its connectivity with other intrinsic networks under consideration, the SN appeared to be highest in the hierarchy, suggesting that it may play an important role in switching anticorrelated networks. These findings add to our understanding of functional brain architectures from the perspective of directed connectivity.

### Functional Architecture of Resting State Networks

A fundament of brain organization is the integration of functionally segregated brain regions (Park and Friston 2013). Several resting-state functional connectivity studies have shown that endogenous activity in the brain is self-organized and highly structured (Biswal et al. 1995; Raichle et al. 2001; Greicius et al. 2009; Roy et al. 2009). Therefore, we had prior expectations about the nature of the functional connectivity between regions in this study and we hypothesized network structure could be inferred from the data. The inferred connectivity from the resting state fMRI data confirmed our hypothesis that the anticorrelation between the core subsystem of the DN and the salience and DANs are driven by asymmetric effective connectivity. The regions within each resting state network showed stronger positive (excitatory) connectivity between each other; such that they clustered into a network. It is interesting to note that the clustering of regions based on effective connectivity was identical to that based on functional connectivity. This similarity provides evidence that the functional dissociation between these networks—that is, often observed with data driven methods like ICA and functional connectivity—reflects the interaction of the hidden neuronal states.

#### Effective Connectivity Within the cDN

Several previous studies have investigated the functional architecture of the cDN during rest using effective connectivity (Li et al. 2012; Di and Biswal 2014; Razi et al. 2015; Sharaev et al. 2016). Although there are inconsistencies in the direction and valence (excitatory or inhibitory) of individual connections across these studies (see Supplementary Fig. S5), they suggest a tight functional coupling within the cDN. This is consistent with our findings. Note the effective connectivity among regions can depend on whether other regions are included in the network (because effective connectivity can be mediated vicariously or polysynaptically via nodes outside any subgraph). Hence, it is difficult to compare the specific connections in our study with quantitative results from different subgraphs; for example, the nodes of 3 core networks in this study versus the DN nodes considered in most previous studies. Furthermore, there are differences in model assumptions, for example, spectral DCM in the current paper and (Razi et al. 2015; Sharaev et al. 2016), versus stochastic DCM in Li et al. (2012) and Razi et al. (2015); and deterministic DCM in Di and Biswal (2014). Further differences between studies also related to second (group) level analysis methods; for example, PEB in this study, versus classical statistics in previous studies. Given these differences between present and previous studies, it is remarkable that several aspects of effective connectivity replicate across studies; including the connection from the left angular gyrus to the PCC, the bilateral angular gyri to the MPFC, and bidirectional connection between the left and right angular gyri. These findings suggest a pattern of functional coupling within the cDN that transcends modeling assumptions, where the angular gyrus may have a driving or modulating role (Sharaev et al. 2016). Importantly, this pattern is conserved when other regions are added to the DCM, as exemplified in our study. This stable participation of the angular gyrus in functional integration echoes its functional role in domain-general automatic processing, functioning as an automatic buffer of incoming information (Humphreys and Lambon Ralph 2017).

#### Effective Connectivity Within the SN

In order to obtain a comprehensive understanding of the functional architecture of the SN, we focused on 5 cortical areas within this network. We found that these 5 regions showed strong bidirectional positive (excitatory) connectivity among each other; with an exception of the connections related to the left anterior PFC. Previous studies, which focused only on the bilateral AI and dACC, found directed extrinsic (between region) connections among these 3 regions during an attention-demanding task (Ham et al. 2013) and a perception decision-making task (Lamichhane and Dhamala 2015). Our finding suggests that there is bidirectional connectivity between these regions, even during rest. This finding is also consistent with the evidence for white matter connections between the 3 regions in the SN (van den Heuvel et al. 2009; Bonnelle et al. 2012). Additionally, we also included the anterior part of the prefrontal cortex (aPFC) in our SN. This region (BA9) is where the von Economo neurons are found (Fajardo et al. 2008) outside the ACC and insular cortex in human (Butti et al. 2013; Morel et al. 2013). This similarity in cytoarchitecture may be the neuronal basis for the strong positive connections among these regions. The stronger connections in the direction from dACC and AI to the aPFC, in relation to their reciprocal connections, is compatible with the differences in functional roles of these regions: dACC and AI support a basic domain-independent and externally directed “task mode”; whereas the aPFC may convey domain-specific task-set signals (Dosenbach et al. 2006, 2007).

#### Effective Connectivity Within the DAN

For the DAN, we found bidirectional positive connectivity between the 6 constituent regions with few exceptions. In a previous study—focusing only on the FEF and IPS—extrinsic connections between the FEF and the IPS and the interhemispheric connections were found during an attention task (Vossel et al. 2012). Our study provides evidence that the directed connections within the DAN are also prominent during rest. The higher connectivity between the FEF and IPS might reflect the pronounced anatomical connectivity between these areas (Umarova et al. 2010). This may imply that attentional interactions between these regions are still in play during rest.

### Hierarchical Organization of the Resting State Networks

The main contribution of the current study is to clarify the relationship between the well-known anticorrelated modes in terms of their causal interactions. Effective connectivity allowed us to probe the asymmetric architecture of these network interactions, which is not possible with symmetric measures of functional connectivity. We found that the salience and the DANs inhibit the activity of the cDN, whereas the cDN exerts a weak excitatory influence on the activity of both of the 2 networks. Our findings are supported by previous literature. For example, in the present study, we show that the cDN regions receive afferent information from most of the other modeled brain regions, whereas the regions in the salience and DANs send efferent information to the rest of the brain. This has also been reported in another study (Yan and He 2011); in which the driven role of the regions in DN and the driving role of the regions in the salience and DANs were suggested by both Granger causality and graph theoretical analysis, although the interpretability of Granger causality analysis is questionable when applied to fMRI data (Smith et al. 2011; Webb et al. 2013). In addition, we found that aPFC exerts no inhibitory influence on the activity of MPFC, which is in line with a recent study that used transcranial magnetic stimulation to excite or inhibit a prefrontal node and found no evidence for the effect of aPFC on spontaneous activity in MPFC (Chen et al. 2013a).

In this work, we focus on the neurobiological implications of our findings in the context of the 3 resting state networks that we set out to examine. A key architectural principle of the brain is its hierarchical organization (Friston 2008), which has been established most thoroughly in the visual system, where lower (primary) areas receive sensory input and higher areas adopt a multimodal or associational role (more discussion, please see Friston 2008). The hierarchical organization of the prefrontal cortex has also been established (Nee and D’Esposito 2016). This neurobiological notion of a hierarchical organization rests upon the distinction between 3 types of extrinsic connections: forward connections which link a lower area to a higher area, backward connections which link a higher to a lower area, and lateral connections that link areas at the same level (Felleman and Van Essen 1991; Bastos et al. 2012).

In the predictive coding framework—which is based upon evidence from cortical hierarchies (Friston 2010)—backward connections deliver predictions to lower levels, whereas forward connections convey prediction errors to the upper levels (Park and Friston 2013). Crucially because backward connections convey predictions—which serve to explain and thereby reduces prediction errors in lower levels—their effective (polysynaptic) connectivity is generally assumed to be inhibitory (Bastos et al. 2012). Furthermore, the hierarchy in the associative cortices can be characterized by the notion that greater efferent (outward) relative to afferent (inward) connectivity reflects a larger influence of one region over another (Badre and D’Esposito 2009; Goulas et al. 2014; Nee and D’Esposito 2016). Consistent with these definitions of the hierarchical organization, Figure 2*C*, which summarizes our main findings, shows average efferent and afferent connectivity for each network, and is clearly indicative of a hierarchical pattern in the 3 networks. The hierarchy score monotonically decreased from (the SN: 0.12, the DAN: 0.07 the cDN: −0.19) suggesting that both the SN and the DAN rank higher than the cDN in this system. It remains to be clarified that the “hierarchy” presented here is a relationship between intrinsic networks, characterized by the differences between efferent and afferent connectivity strength. The higher ranking only means greater efferent–afferent difference, rather than higher ranking in cortical or functional hierarchy.

This hierarchical pattern echoes the previous observation that the rAI, a region in the SN, is a causal driving hub in a system including the salience, default and central–executive networks, suggesting its critical and causal role in the initiation of spontaneous switching between the default and the central–executive networks (Sridharan et al. 2008; Uddin 2015). This finding is also supported by evidence from fMRI studies of patients with traumatic brain injury within the SN. These studies showed that integrity of white-matter tracts within the SN is necessary for causal influence of the SN on the activity of the DN (Bonnelle et al. 2012; Jilka et al. 2014). One possible physiological basis—for the hierarchical location of the SN—may rest on a unique neuronal cell type; namely, the von Economo neuron (VEN) that is exclusively localized to the dACC, AI and aPFC (Fajardo et al. 2008; Butti et al. 2013; Morel et al. 2013). The particular dendritic architecture of VENs enables it to play the role of a rapid relay to the other parts of the brain and has been associated with the anticorrelated networks (Williamson 2007; Sridharan et al. 2008). Additionally, VENs are found in the deeper cortical layer (layer V), which is where backward connections arise (Friston 2008). From the perspective of predictive coding, the precision estimated in the SN could be understood as an attention to ascending prediction errors that informs higher level representations of self-generated thoughts related to construct personal meaning from salient information which are subserved by the cDN (Andrews-Hanna et al. 2014). The precise detail of the physiological mechanisms by which the ensuing anticorrelation is mediated is an interesting focus for future work.

### From Effective Connectivity to Functional Connectivity

To obtain further insight into the casual interactions between these regions in the anticorrelated networks, we also report the functional connectivity, summarized as correlations between each pair of nodes. As highlighted in Figure 3*E*, functional connectivity between the right AI and the right IFG is relatively strong; however, the effective connectivity analysis (Fig. 3*B*) revealed the asymmetric nature of this connection. Only the connection from the right AI to right IFG is evident, whereas the reciprocal connection is absent. This sort of asymmetry, afforded by DCM, cannot be identified using symmetric functional connectivity measures. Besides this asymmetry, other differences can be seen when comparing the 2 connectivity mappings in Figure 3*B*, *E*. An example is in the connection with significant functional connectivity (e.g., left FEF to left IFG), where effective connectivity is absent (indicated by an arrow). These differences can be explained by what the respective connectivity measures characterize. Functional connectivity is essentially a summary of the data, computed as pairwise correlations that reflect statistical dependencies among regional measurements. In contrast, effective connectivity, as computed by DCM, reflects the neuronal interactions that induce the BOLD response and, in turn, the functional connectivity. Another possible source of spurious functional connectivity is observation noise. DCM explicitly estimates additive (Gaussian) observation noise and separates this from the estimates of effective connectivity, whereas functional connectivity analysis cannot make this distinction.

### Limitations

This study has several potential limitations. We selected key nodes or regions to understand the anticorrelation between networks, which is motivated by the specific relationship between the core DN and the salience and DANs. Considering the heterogeneity of anatomy and function of the DN (Damoiseaux et al. 2008; Andrews-Hanna et al. 2010b; Yeo et al. 2011), especially the heterogeneity in anticorrelation found recently (Chen et al. 2017; Dixon et al. 2017), it is possible that heterogeneity of the DN is reflected in effective connectivity, which could be investigated in future. In addition, we did not consider other networks (or modes), such as the central executive network (CEN), which is—like the SN—an important component of a frontoparietal system (Spreng et al. 2010, 2013). Previous work suggests that the frontoparietal system is interposed between the default and DANs and may mediate their interactions (Vincent et al. 2008; Spreng et al. 2013). It has also been suggested that the SN plays a switching role between the central executive and default mode networks (Sridharan et al. 2008; Goulden et al. 2014). In terms of well-known anticorrelation patterns, the connectivity between the CEN and the DN is not entirely clear (such as Raichle 2011). From the perspective of whole-brain networks, future studies could extend the current study by including the CEN and/or other networks such as sensorimotor network, visual network and auditory network in the DCM—to explore how these networks interact with each other and ask whether there is any functional specialization (within the frontoparietal system) in mediating the interaction between the default and DANs. This is a promising future direction; especially when armed with computationally efficient and accurate procedures (Razi et al. 2015) for fitting whole-brain DCMs (Razi et al. 2017).

In addition, it should be noted that these findings were obtained from healthy adolescent and young adults (age range: 14–23 years) and thus may not generalize to the general population. We were interested in this age range because approximately half of the lifetime burden of mental illness starts by age 14 years and around 75% of mental illnesses have an onset prior to age 24 (Kessler et al. 2005). Thus the present findings may provide a reference for future studies on understanding the neurodevelopmental basis of mental illnesses. However, we acknowledge that the brain connectivity is still developing in the age range of our cohort—and undergoes changes across the lifespan (Richmond et al. 2016; Zuo et al. 2016). Whether the hierarchical organization discovered in this report changes across lifespan is an interesting question that will require further study.

Finally, we note that the current study restricted itself to studying the characteristics of effective connectivity that were conserved across participants. In future work, we will explore the genetic and environmental contributions to large-scale network architectures described above, using our twin data.

## Conclusion

The current study provides a mechanistic insight into how the regions in anticorrelated intrinsic brain modes interact and suggests a causal role of the SN in modulating descendant modes in a hierarchical setting. These findings help us understand the causal processes among key resting state networks that maintain normal mental states (e.g., arousal) and cognition (e.g., working memory and executive functions) during development and aging (Chai et al. 2014; Keller et al. 2015; Spreng et al. 2016; Wang et al. 2016). Any disruptions to the implicit modulation or contextualization of distributed processing may lead to psychopathology in several neurological and psychiatric disorders, such as attention deficit and hyperactivity disorder, schizophrenia and dementia (Williamson 2007; Jiang et al. 2013). More generally, our study illustrates the power of DCM in discovering networks and understanding the mechanisms of brain network organization. This application of the newly developed framework of Bayesian model reduction and empirical Bayes to the resting state-fMRI time series opens a new avenue for investigating the effective connectivity of networks with a large number of candidate regions using graph theoretic analysis (Rubinov and Sporns 2010).

## Supplementary Material

Supplementary Data
